# Activated ovarian endothelial cells promote early follicular development and survival

**DOI:** 10.1186/s13048-017-0354-z

**Published:** 2017-09-19

**Authors:** Alon Kedem, Anate Aelion-Brauer, Peipei Guo, Duancheng Wen, Bi-Sen Ding, Raphael Lis, Du Cheng, Vladislav M. Sandler, Shahin Rafii, Zev Rosenwaks

**Affiliations:** 1 The Center for Reproductive Medicine and Infertility, Weill Cornell Medical College, New York, NY 10021 USA; 2 Ansary Stem Cell Center for Regenerative Medicine at Weill Medical College of Cornell University, New York, USA

**Keywords:** Endothelial cells, in-vitro maturation, Follicle culture, Follicular activation

## Abstract

**Background:**

New data suggests that endothelial cells (ECs) elaborate essential “angiocrine factors”. The aim of this study is to investigate the role of activated ovarian endothelial cells in early in-vitro follicular development.

**Methods:**

Mouse ovarian ECs were isolated using magnetic cell sorting or by FACS and cultured in serum free media. After a constitutive activation of the Akt pathway was initiated, early follicles (50–150 um) were mechanically isolated from 8-day-old mice and co-cultured with these activated ovarian endothelial cells (AOEC) (*n* = 32), gel (*n* = 24) or within matrigel (*n* = 27) in serum free media for 14 days. Follicular growth, survival and function were assessed.

**Results:**

After 6 passages, flow cytometry showed 93% of cells grown in serum-free culture were VE-cadherin positive, CD-31 positive and CD 45 negative, matching the known EC profile. Beginning on day 4 of culture, we observed significantly higher follicular and oocyte growth rates in follicles co-cultured with AOECs compared with follicles on gel or matrigel. After 14 days of culture, 73% of primary follicles and 83% of secondary follicles co-cultured with AOEC survived, whereas the majority of follicles cultured on gel or matrigel underwent atresia.

**Conclusions:**

This is the first report of successful isolation and culture of ovarian ECs. We suggest that co-culture with activated ovarian ECs promotes early follicular development and survival. This model is a novel platform for the in vitro maturation of early follicles and for the future exploration of endothelial-follicular communication.

**Capsule:**

In vitro development of early follicles necessitates a complex interplay of growth factors and signals required for development. Endothelial cells (ECs) may elaborate essential “angiocrine factors” involved in organ regeneration. We demonstrate that co-culture with ovarian ECs enables culture of primary and early secondary mouse ovarian follicles.

## Background

Advancements in cancer treatment continue to improve survival and cure rates in women of reproductive age. Many, however, will struggle with ovarian failure and premature menopause as a consequence of potentially gonadotoxic chemotherapy and radiation [1]. Among the options currently available for fertility preservation in these patients is cryopreservation and future auto-transplantation of ovarian cortical tissue containing immature follicles [2, 3]. While excellent progress has been made in this clinical arena, efficiency of such a technique remains compromised secondary to a period of hypoxia following grafting [4–6]. Additionally, this technique carries the risk of potentially reintroducing malignancies [7]. These drawbacks could be overcome by the ability to mature early follicles in vitro. The development of this approach is currently hampered by the lack of knowledge regarding signals responsible for early follicle activation. Duplicating the complex interaction of growth factors, cellular and hormonal signals required for follicular development and oocyte maturation in vitro is challenging.

During embryogenesis, endothelial cells (ECs) induce organogenesis before the development of circulation [8–11]. These findings suggest that ECs not only serve as conduits to deliver nutrients and oxygen, but may also be instrumental in establishing an instructive, organ-specific roadmap through which elaboration of paracrine trophogens stimulate regeneration. These elaborate signals promoted by ECs have been termed “angiocrine factors” and have been shown to be crucial for the maintenance of organ-specific tissues and tumor cells [12]. ECs contribute to self-renewal of hematopoietic stem cells [13–15] and neuronal stem cells [16]. They promote the growth of leukemic cells [17], gliomas [18] and more recently, liver and lung regeneration have been found to be dependent on specific angiocrines [19]. Angiogenesis has also been shown as critical in the process of early follicular development. Angiogenesis begins within the stroma during early follicular development [20]. Primordial and primary follicles receive their nutrition and oxygen supply via passive diffusion from stromal blood vessels. Stromal cells that surround follicles in the secondary stage or later become organized into thecal layers, in which the innermost part contains blood vessels. This vascular system is thought to provide various paracrine factors necessary for folliculogenesis, most notably VEGF [21]. However, hurdles associated with cultivating functional primary endothelial cells in long term in vitro culture systems have hindered their use. To bypass this problem, Seandel and Rafii et al. demonstrated that primary endothelial cells can be transduced with an adenovirus gene, early region 4 encoded open reading frame-1 (E4ORF1), which leads to chronic activation of Akt, thereby blocking apoptosis and enabling culture of primary endothelial cells for weeks, while maintaining their angiogenic profiles [22]. Incorporating primary mouse ovarian Akt-activated ECs, we propose a novel, serum free, in vitro follicle growth system that promotes activation and development of early follicles by providing follicles with appropriate angiocrines, at the appropriate time, and maintains the essential communication between ECs and follicles.

This study aims to analyze the distinct characteristics of ovarian endothelial cells and to apply this novel platform as an in-vitro system to induce follicular maturation for fertility preservation.

## Methods

### Animals

All animal procedures performed in the study were reviewed and approved by the Institutional Animal Care and Use Committee. C57BL/6 (B6) mice were used in this study.

### Isolation and culture of ECs from murine ovaries

Six-week-old B6 female mice were euthanized, and their ovaries surgically removed with sterile technique. Ovaries were placed in HBSS supplemented with 1% Penicillin/Streptomycin/Amphotericin B solution (Life Technologies, Norwalk, CT) and minced with surgical scissors. Minced ovarian tissue was re-suspended in HBSS solution, spun down and supernatant discarded. Tissue was digested with Collagenase A (Roche, Nutley, NJ) at a concentration of 2.5 mg/ml for 45 min at 37 °C with constant, gentle tilting. Once fully digested, ovarian tissue was filtered through a sterile 40um cell strainer. The flow-through was centrifuged at 1200 RMP for 5 min and supernatant carefully aspirated. Room temperature dilute lysis buffer (BD Pharmingen Franklin Lakes, NJ) was used for red blood cell lysis. Cells were then counted using a grid.

### Isolation of murine ovarian ECs with magnetic bead sorting

Sheep anti-rat Dyna beads (Invitrogen Carlsbad, CA) were coated with rat anti-mouse CD31 antibody, a known endothelial cell marker (Ten μl of rat anti-mouse CD-31 antibody per 10 μl beads) (Clone 13.3, BD Pharmingen) as per manufacturer protocol. Ten microliters of beads were used per 10 ovaries. Beads were incubated overnight at 4 °C. When ready for use, beads were washed three times with PBS, 0.1% BSA, 2 mM EDTA and 1% Penicillin/Streptomycin/Amphotericin B solution (Life Technologies) to remove excess antibody. Beads were re-suspended in 50 μl of the above washing solution until they were ready for use.

Ten microliters of CD-31 coated beads were added per 1 × 10^6^ cells counted. The cells were incubated for 45 min at 4 °C with constant, gentle tilting. After incubation, bead bound cells were collected with a magnetic particle concentrator (Invitrogen Norwalk, CT) and washed five times with PBS + 0.1%BSA + 2 mM EDTA + 1% Penicillin/Streptomycin/Amphotericin B (Life Technologies).

### Isolation of murine ovarian ECs using fluorescence activated cell sorting

Twenty-five micrograms of VE-Cadherin (BV13, Biolegend San Diego, CA)-Alexa Fluor 647 was injected retro-orbitally under anesthesia 8 min prior to sacrifice and organ harvest. For flow sorting, ovaries were minced and incubated with Collagenase A (25 mg/ml), Dispase II (25 mg/ml), and DNase (250 μg/ml) (Roche) at 37 °C for 20–30 min to create a single cell suspension. Cells were filtered through a 40 μm filter immediately prior to analysis. Post digestion staining was performed with CD31 and non-endothelial antibodies CD45 (30-F11, BD Pharmingen), rat and mouse IgG (Jackson Laboratories, Farmington, CT). All flow cytometry was performed on a LSRII SORP. All flow sorting was performed on an AriaII SORP. Data analysis was done with BD FACS Diva software (Becton Dickenson). Antibody captured beads were used to calculate compensation (BD Pharmingen). FSC-H vs. FSC-W and SSC-H vs. SSC-W were analyzed to exclude cell doublets. All manufacturers recommended quality control tests were performed immediately prior to sorting.

### Ovarian EC culturing

Primary ovarian ECs (POECs) were re-suspended in 500ul of EC growth media containing Nutrient Mixture F12 Ham (Sigma-Aldrich St Louis, MO), Low glucose DMEM (Sigma-Aldrich), Penicillin/Streptomycin/Amphotericin B solution (Life Technologies), MEM Non-Essential Amino Acids (Life Technologies), 1 M HEPES (Life Technologies), KnockOut™ Serum Replacement(Life Technologies), 10 mg/ml heparin stock (Sigma-Aldrich), 7.5 mg/ml endothelial cell mitogen (Biomedical Technologies Stoughton, MA) and transferred to fibronectin (Sigma-Aldrich) coated plates.

On day 7 of culture, POECs were transduced with myristoylated-AKT1 (myr-AKT) lentiviruses as previously described [15].

Flow cytometry purity confirmation was done after 6 passages. Cells were stained with VE-Cadherin, CD31, CD45 and Dapi (Sigma-Aldrich). All cells were blocked prior to staining and analyzed using a LSRII SORP (BD Biosciences).

### Angiocrine profiling of ovarian endothelial cells

Total RNA was extracted from POEC (isolated by FACS) by the Mini RNA Isolation I Kit (Zymo Research Corp., Irvine, CA), according to manufacturers’ protocol. Total RNA (100 ng) from each sample was used for cDNA synthesis using high capacity real-time (RT)-PCR Kit (Applied Biosystems, Carlsbad, CA) according to manufacturer’s protocol: 37 °C for 120 min followed by 85 °C for 5 min with random hexamer primers (25 ng/μl final concentration) in a 10 μl total volume reaction.

RT-PCR reaction mix contained cDNA, Fast SYBR Green Master Mix (Applied Biosystems) and specific primers for VE-cadherin, FLK1, VEGF-A, PDGF, FGF1, VWF, Notch1, Notch2, Notch3, Notch4, Dll1, Dll4, Jagged1, Jagged2, and β-actin (endogenous control) in a total volume of 10 μl Reaction was performed in a StepOne plus RT-PCR apparatus (Applied Biosystems). Cycling parameters were 20 s at 95 °C, 40 cycles at 95 °C for 3 s and at 60 °C for 30 s. A melting curve analysis was performed at the end of each run to ensure a single amplicon. Analysis of qRT-PCR results was carried out using StepOne software (Applied Biosystems). Relative gene expression was calculated using the delta-delta Ct method.

### Murine ovarian follicle isolation

Intact ovaries from euthanized 8-day-old B6 mice were removed under sterile conditions and immediately placed in warmed dissecting media composed of Waymouth MB752 (Sigma-Aldrich) supplemented with Penicillin/Streptomycin/Amphotericin B solution and 10% Fetal Bovine Serum (Life Technologies). Oocyte-granulosa complexes were mechanically isolated using insulin gauge syringes under a dissecting microscope. Intact complexes measuring 50-150 μm were washed, isolated and, with drawn glass micropipettes, transferred to wells containing one of three culture platforms.

### Follicular culture

Individual follicles were cultured for 14 days on one of the following platforms: 1) AOEC + follicular culture media (*n* = 32), 2) Matrigel + follicular culture media (*n* = 27), 3) Gelatin + follicular culture media (*n* = 24). Experiments were run in parallel (Fig. 3a).

All experiments were carried out in 48-well plates. AOEC co-culture plates were prepared by first coating plates with fibronectin. Briefly, 0.5 ml of dilute fibronectin (Sigma-Aldrich, diluted 1:1000 in PBS) was added to plates. Plates remained at room temperature for 30 min after which solution was removed by aspiration. Plates were then seeded with 0.5 million endothelial cells per plate. Matrigel (BD Biosciences) plates were prepared as previously described with slight modification [23]. Briefly, Matrigel was liquefied at 4 °C and diluted in a 1:1 ratio with follicular culture media. Plates were then maintained at 37 °C for one hour to allow for polymerization. Gelatin (Sigma-Aldrich) plates were coated as per manufacturer protocol. 200uμl of follicular culture media was added to all plates.

The individual placing the follicles into the various wells was blinded to platform. Half of the media from each well was exchanged for fresh media every second day. Culture media included: Waymouth MB752 supplemented with 0.23 mM pyruvic acid (Sigma-Aldrich), Penicillin/Streptomycin/Amphotericin B solution (Life Technologies), 10mIU/ml recombinant FSH (Merck, Whitehouse Station, NJ), 5 μg/ml each of insulin, transferrin and selenium (ITS, Becton Dickinson), 1 mg/ml fetuin (Sigma-Aldrich) and 10% KnockOut™ Serum Replacement (Life Technologies). Throughout the 14 days of culture, oocyte-cumulus complexes were maintained at 37 °C in a Forma™ Series 3 Water Jacketed CO2 Incubator with 5% CO2 and humidified air.

### Follicular and oocyte measurements

Each individual follicle was observed and photographed every second day using a Nikon-2000 U inverted microscope with digital imager. All photographs were imported into ImageJ software (version 1.48, National Institutes of Health) for measurement. Two perpendicular diameters of follicles were measured. The mean of two diameters was calculated and considered as the reference diameter of the follicle. Diameters of oocytes were taken in the same fashion. The first day of culture was considered day 0. Follicular atresia was characterized by absence of growth, a decrease in diameter and extrusion of the oocyte.

### Estradiol measurements

In order to obtain potential hormonal measurements, we cultured 5 secondary follicles (100–150 μm) in each well. ½ media was collected and replaced every second day. This substituted media was stored individually in eppendorf tubes at -20 °C. Estradiol measurements were analyzed using DPC immulite 2000 immunoassay system (Seimens Healthcare, Tarrytown, NY). Media from wells containing no follicles was used as controls.

### Isolation of Mouse Tail Fibroblasts (MTF)

Tail and ear biopsies were taken from 6-week-old B6 mice under sterile conditions. Tissue was minced and incubated at 37° for 45 min in trypsin-EDTA (Life Technologies). The trypsin was deactivated with 20% fetal bovine serum (FBS) in DMEM (Gibco/Life Technologies) with 1% Penicillin/Streptomycin/Amphotericin B solution (Life Technologies). After 48 h the media was replaced with 10%FBS in DMEM with 1% Penicillin/Streptomycin/Amphotericin B solution and the cells were allowed to expand for 10–14 days.

### TUNEL and Ki67 staining

In order to detect apoptosis in oocytes and granulosa cells, follicles co-cultured on AOECs or MTF for 7 days were analyzed for DNA fragmentation with TUNEL reaction. TUNEL reaction was performed according to manufacturer’s labeling protocol for adherent cells (Roche).

In order to detect markers of proliferation in granulosa cells on day 7 of co-culture with AOECs or MTF, follicles from each group were stained with Ki67 (stain for proliferating cells) according to manufacturer’s protocol (abcam Cambridge, MA).

All cells were photographed on a Zeiss LSM 710 microscope equipped with epifluorescent optics.

### Presentation of data and statistical analysis

Two Groups were created for analysis: follicles < 100 μm (Primary follicles) and follicles > 100 μm (secondary follicles). Follicular growth between groups is presented as difference in mean diameter of follicle compared to day 0 of culture. Analysis of variance was used for comparison of means. The Kaplan Meier estimate and log-rank test were used to compute and compare survival over time between all three groups. All statistics were computed with Stata12 software. Statistical difference was defined as *p* < 0.05.

## Results

### Ovarian endothelial cell isolation and creation of serum-free culture system

**Fig. 1:**
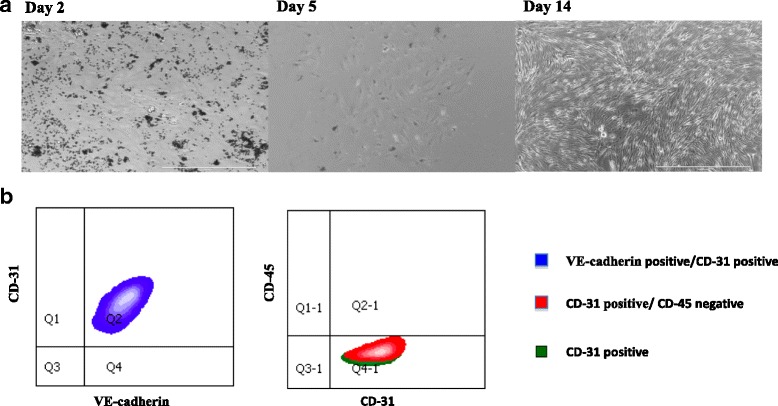
Isolation and Culture of Primary Ovarian Endothelial Cells. **a**. Mouse ovarian ECs (CD31+) were isolated using magnetic cell sorting and cultured in serum free media. 7 days later, activation of the ECs was initiated with lentivirus transduction of the myr-Akt gene. 14 days post isolation, AOEC are proliferating. **b.** FACS analysis of AOEC isolated by magnetic cell sorting, cultured on serum free media, passage 6. 98% of the cells were CD 31 positive, VE-Cadherin positive and CD45 negative, matching a known EC profile. This experiment was repeated 5 times; all cultures contained at least 93% fully matched endothelial cells

Two approaches were tested for isolation of ovarian endothelial cells: Immunomagnetic sorting and FACS. Using conventional monoparametric labeling with magnetic particles for isolation, we established a CD31 positive cell population from B6 mouse ovaries. An average of 2% CD31 positive cells were acquired (1 × 10^4^-3 × 10^4^ cells per mouse) per isolation experiment. After 6 passages all cells were scanned by FACS. On average, 93% of cells were CD 31 positive, VE-Cadherin positive and CD45 negative (Fig. 1b).

We established a second, high fidelity approach to purify and immediately profile ECs from an in-vivo source by FACS. After intra-vital staining with VE-cadherin, and post digestion staining with CD31 and CD45, an average of 3% VE-cadherin positive, CD31 positive and CD45 negative cells were obtained (1 × 10^4^-2 × 10^4^ cells per mouse). After 6 passages all cells were scanned by FACS. On average, 95% of the cells were CD 31 positive, VE-Cadherin positive and CD45 negative, superior purities compared to magnetic isolation.

Primary endothelial cell cultivation is a great challenge. In our experience, non-activated POEC could not last more than two passages, insufficient for any in vitro co-culture studies. To circumvent this problem without adding growth factors that may confound follicular growth, POECs were transduced with an adenovirus gene, myr-Akt, which leads to constitutive activation of Akt and enables co-culturing of POEC with follicles in serum and growth factors-free medium for weeks, while maintaining their ovarian angiogenic signature (Fig. 1a).

### Ovarian Endothelial cell characterization

**Fig. 2:**
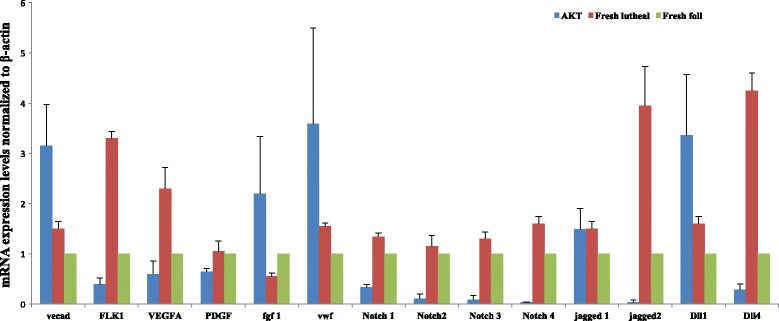
Characterization of cultured, activated ovarian ECs in comparison to fresh ovarian ECs in the follicular and luteal phase. Fresh ovarian endothelial cells were harvested and sorted by FACS before (Foll) and after (Luteal) ovulation. They were compared to AOED (AKT). Relative mRNA expression of endothelial cell markers were investigated

Relative mRNA expression of endothelial cell markers such as VEGF-A, VWF and VE-Cadherin further confirm that our AOECs match a known EC phenotype (Fig. 2).

In comparing AOECs to fresh ovarian ECs from follicular and luteal phases of the estrous cycle, endothelial cell markers had comparable expression in AOECs relative to the fresh groups. Relative expression of selected angiocrines that may be involved in early folliculogenesis such as VEGF-A and Notch ligand jagged-1 were relatively comparable in AOEC and fresh ECs. Other angiocrines such as VE-Cadherin, FGF-1, VWF and Dll-1 were relatively up regulated in the AOEC group (Fig. 2).

### Follicular and oocyte growth in culture systems

**Fig. 3:**
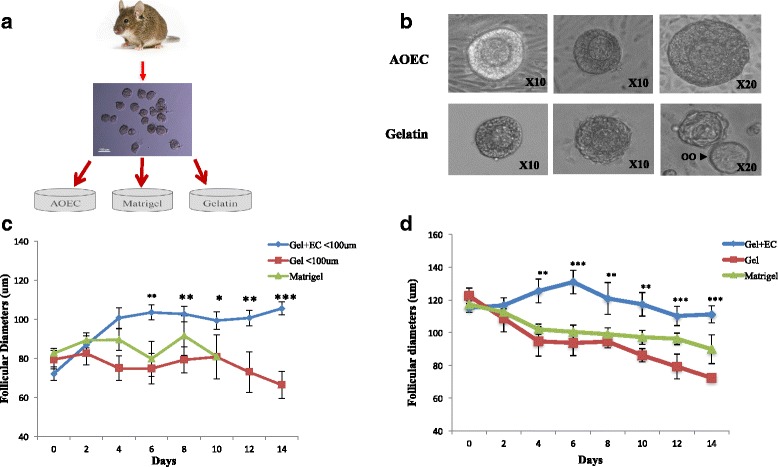
Early follicular culture in serum free conditions. Each individual follicle was observed and photographed every second day. Two perpendicular diameters of follicles were measured. The mean of two diameters was calculated and considered as the reference diameter of the follicle. Diameters of oocytes were taken in the same fashion. The first day of culture was considered day 0. **a.** Early follicles (50–150 μm) were mechanically isolated from 8-day-old mice and co-cultured with AOECs (*n* = 32), Matrigel (*n* = 27), or gel (*n* = 24) in serum free media for 14 days. **b.** Early follicles cultured on AOECs versus gelatin on days 2, 6 and 14. Growth and proliferation seen in AOEC group versus progressive degeneration and eventual oocyte (oo) extrusion in gelatin group. **c.** Follicular growth in primary follicles (<100 μm). **d.** Follicular growth of secondary follicles (>100 μm)

Co-culture with AOEC did enhance primary follicular growth in comparison to Matrigel and Gel culture systems (Fig. 3b). Primary follicles co-cultured with AOECs grew an average of 33 μm, a significant increase in growth after day 4 of culture in comparison to the other two groups (Fig. 3c). Following day 10 primary follicles on matrigel collapsed and diameters couldn’t be measured. Secondary follicles grew an average of 15 μm, however, their growth plateaued on day 6 of culture (Fig. 3d). Both control groups had a significant decline in follicular diameter in both follicular classes.

**Fig. 4:**
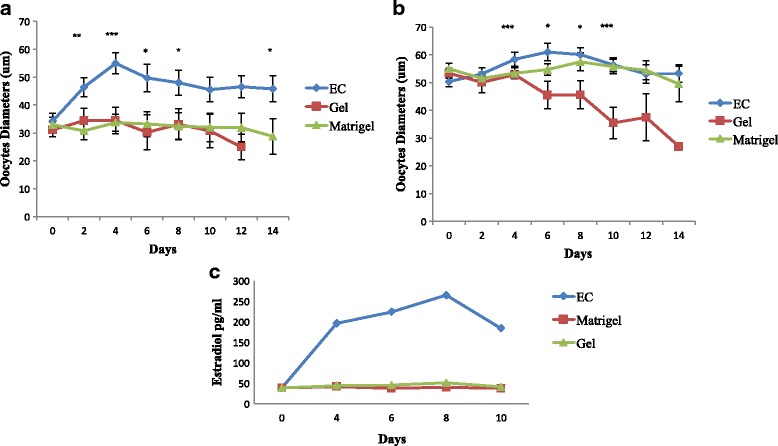
**a.** Oocyte growth and estradiol concentrations. **a** Oocyte growth of oocytes < 45 μm **b**. Oocytes growth of oocytes >45 μm **c.** Follicular estradiol secretion. Due to undetectable levels of Estradiol in primary follicles and from individual follicles, we cultured 5 secondary follicles (100–150 μm) in each well

AOECs also promoted oocyte growth, however, predominantly in primary follicles. Initial oocyte diameters in primary and secondary follicles co-cultured with AOEC were 34 ± 2.7 μm and 50 ± 1.8 μm, with growth of 60 and 20% respectively (Fig. 4a-b). Oocytes from co-cultured secondary follicles had similar diameters as the matrigel group but both were significantly larger than the gel group.

### Follicular survival

**Fig. 5:**
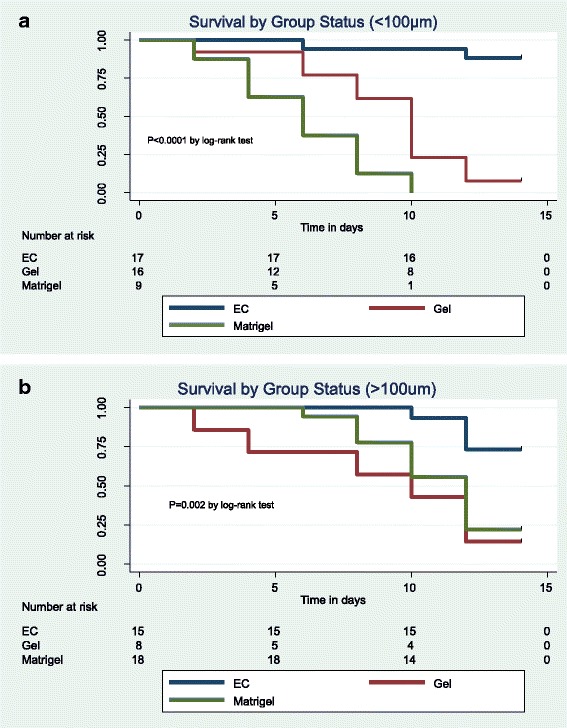
Follicular survival rate. Follicular atresia was characterized by absence of growth, a decrease in diameter and extrusion of the oocyte. **a.** Survival in follicles <100 μm **b.** Survival in follicles >100 μm

AOEC co-culture maintained the survival of primary and secondary follicles throughout the culture period (Fig. 5a-b). After 14 days of culture with AOEC, 73 and 83% of the follicles survived respectively. However, most of the follicles cultured on gel or matrigel groups did not survive (primary follicles 0 and 7%; secondary follicles 12 and 22%, respectively).

### Follicular estrogen secretion

In order to evaluate the capacity of the follicles to secrete hormones, we analyzed growth media every other day of culture for estradiol levels. Due to undetectable levels of Estradiol in primary follicles and from individual follicles, we cultured 5 secondary follicles (100–150 μm) in each well. Increasing levels of estradiol were detected only in media collected from AOEC co-cultured follicles starting day 2 of culture (peak estradiol concentration of 224 ± 48 pg/ml) (Fig. 4c).

### Assessment of apoptosis or proliferation in granulosa cells and oocytes

In an attempt to investigate how AOECs promote in vitro development and survival of early follicles in comparison to co-culture with another cell type, we stained early follicles after 7 days of co-culture with AOEC or MTF with TUNEL (detection of DNA fragmentation) and Ki67 (stains proliferating cells).

**Fig. 6:**
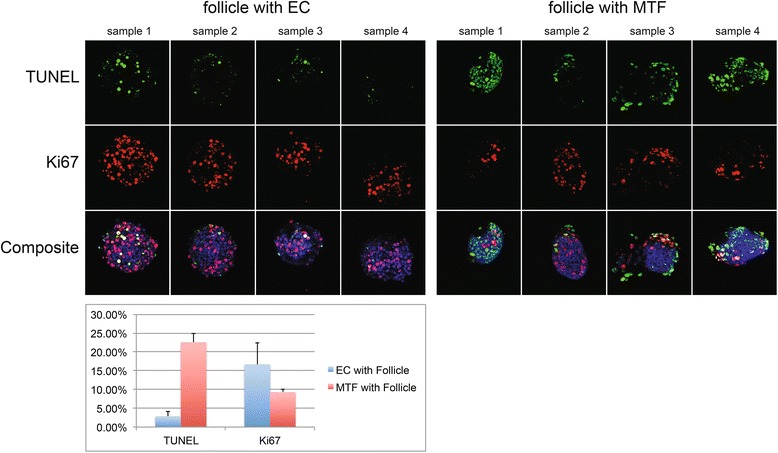
Assessment of follicular apoptosis and granulosa cells proliferation. Early follicles after 7 days of co-culture with AOEC or MTF were stained with TUNEL (detection of DNA fragmentation) and Ki67 (stains proliferating cells)

TUNEL reaction and Ki67 stain showed that follicles cultured on AOEC had significantly less DNA fragmentation and more cells in a proliferative phase than those cultured on MTF (Fig. 6).

## Discussion

It is now evident that the endothelium is more than an inert conduit for blood flow. Tissue-specific endothelial cells, by expressing unique repertoires of trophic growth factors, known as angiocrine factors, support the homeostasis and regeneration of different tissues [10, 15, 18, 19]. This is the first attempt to isolate and explore the distinctive characteristics of ovarian endothelial cells. We established a novel primary mouse ovarian AKT-activated EC culture, in serum free conditions, matching a known EC profile. By co-culturing early follicles with our AOECs, we present a platform with which to interrogate the role of ovarian ECs in early folliculogenesis, as well as enhance follicular growth and survival.

The microvascular circulation of the ovary contains micro-cappilaries that connect arteries to veins and the lymphatic system. Reliable isolation is the first step in establishing a primary ovarian endothelial cell culture system. In this study, two approaches were tested for isolating ovarian endothelial cells: First, conventional monoparametric labeling with magnetic particles was employed. This inexpensive and accessible approach acquired an average of 2% CD-31+ cells with an average 1 × 10^4^ cells per mouse. On average, 10 ovaries were needed in order to isolate a critical mass of ECs for an efferent culture. This fact makes autologous ECs culture almost impossible at this point. These isolations resulted in an average of 93% CD 31 positive, VE-Cadherin positive and CD45 negative cells after 6 passages, making it a suitable and reliable method for production of an ovarian endothelial cell culture system. It should be emphasized that using this method all types of ovarian ECs (artery, vein and lymph) are isolated. Our laboratory, however, has recently established a second, high fidelity approach to purifying and immediately profiling ECs from an in vivo source by FACS sorting after intravital staining with VE-cadherin and post digestion staining with CD31 and CD45 [24]. This technique resulted in superior purities compared to magnetic isolation (on average, 95% of the cells were CD 31 positive, VE-Cadherin positive and CD45 negative) The animals are killed 10 min after infusion of monoclonal antibodies to EC-specific surface markers in order to avoid leakage of antibodies into the lymphatic vessels.. Therefore, in experiments undertaken to profile and characterize purified POECs, FACS isolation was used in lieu of magnetic isolation.

Building a reliable culture system also demands an effective cultivation of POECs that maintains survival and proliferation of the endothelial cells. Previously existing models designed to interrogate the role of endothelial cells in proliferation of solid tumors have been carried out in culture conditions that require supplementation of serum and various growth factors such as VEGFA, FGF2 and EGF which may serve as confounding factors when co-culturing with other cell types [25–27]. In order to circumvent using serum and growth factors, long-lasting endothelial cell cultures have been generated by using oncogenic factors including simian virus 40 large T antigen [28], polyoma middle-T [29] and telomerase reverse transcriptase [30]. Propagation of endothelial cells via these approaches results in chronic activation of the MAPK signaling pathway, which leads to generation of highly proliferative endothelial cells that have lost their innate angiogenic features. Evading these hurdles would require a new approach to generation of a stable endothelial cell culture that could propagate without oncogenic transformation or need for additional growth factors [22]. This goal is achieved by transducing primary endothelial cells with the E4ORF1 gene of the adenovirus serotype 5 or 9 or introduction of myr-AKT which results in constitutive activation of AKT but not MAPK [22]. The availability of a robust population of endothelial cells that retain their innate angiogenic features and are maintained without serum or growth factors is critical in the in vitro study of the role of ECs in organogenesis, tumorogenesis and cell growth. In our study, the angiogenic profile of AKT-activated ovarian ECs was similar, but not identical to that of fresh ovarian ECs (Fig. 2). While this may potentially confound follicular growth, currently, there is no more effective way to propagate and maintain an adequate culture of endothelial cells appropriate for co-culture without addition of growth factors.

One concern that could be raised is that the angiogenic profile of AKT-activated ovarian endothelial cells was similar, but not identical to that of fresh ovarian endothelial cells. However, the expression of selected angiocrines that may be involved in early folliculogenesis (VEGF-A and Notch ligand jagged-1) were relatively comparable in AOEC and fresh ECs. In addition, VE-Cadherin, FGF-1, VWF and Dll-1 were even up regulated in the AOEC group. Since ovarian endothelial cells play a central role in neovascularization during follicular development, as demonstrated in mice [31], the comparable expression of angiocrines between AOEC and fresh EC is essential.

Follicle culture systems for mouse [32–34], large animals [35, 36] and humans [37] have been developed in both 2-dimensional (2D) and 3-dimensional (3D) formats. These systems have been successful in supporting the development of primary and early secondary follicles, but have not yet been adapted for routine use due to several limitations. One hurdle in establishing an effective IVM system is determining an optimal approach to primordial follicle activation/recruitment. The primordial follicle contains an oocyte arrested in the dictyate stage of meiosis, a single layer of squamous granulosa cells and a basal lamina, creating a microenvironment in which the granulosa cells and oocyte co-exist [38]. Paracrine communication between the oocyte, its granulosa cells, adjacent thecal cells and surrounding follicles all combine to control primordial follicle recruitment [38]. This communication is largely mediated by secreted growth factors including several members of the transforming growth factor-beta (TGFβ) superfamily [39]. However, the role of the vascular niche in early follicular activation and development has yet to be investigated. In order to properly investigate the role of a vascular niche in early follicle activation, one would ideally create a model of isolated primordial follicles grown in co-culture with endothelial cells. However, we found mechanical isolation of intact primordial follicles very challenging. Enzymatic isolation also proved challenging, as follicles rarely retained their intact structure. Several studies have circumvented this challenge by creating a two-step culture system where by primordial follicles are grown in a slice of intact ovary and then isolated once they reach the primary or early secondary phases [40, 41]. Since our group was interested in intimate and direct communication between follicles and endothelial cells, this method of culture was not suitable.

Another challenge of IVM is re-creating a 3-D environment in which cell-cell and cell-matrix interactions can efficiently take place. Historically, non-spherical (2-D) culture systems using gel-coated dishes or membranes coated with extracellular matrix proteins were developed with excellent results, including the growth of primary follicles to complete maturation, fertilization and developmental competence [32, 33, 42]. However, follicles cultured in 2-D systems only partially maintained their spatial configuration and relationships, letting the follicular cells expand at the bottom of the dish, with the consequence of potential loss of oocyte-follicular cell interactions. To address this issue, spherical (3-D) culture systems have been developed including matrigel [23], collagen matrix [43], calcium alginate matrix [44] and hyaluronan hydrogel [45]. In choosing controls for this study, we chose gel coated wells for a 2D control and Matrigel for a 3D control, as both are commonly used and approachable systems for follicle culture. We chose not to incorporate our AOECs into a 3D culture, as this would have presented an additional confounding factor in follicular development and interaction with the ECs.

Despite existing in a 2D system, we found that a system incorporating co-culture on AOECs was superior to both the 2D gel system and the 3D matrigel system in maintaining survival and growth of primary and early secondary follicles. Previous reports of 2-D culture systems have described pre-antral follicle survival rates of 48–68% [23, 46, 47] We achieved rates as high as 73% in co-cultured follicles <100um and 83% in co-cultured follicles >100um. These were significantly higher than survival rates in either control group (Fig. 5a, b).

Co-cultured primary follicles showed expeditious growth between days zero and six with a plateau thereafter. Co-cultured secondary follicles showed a milder increase. This observation suggests that factors secreted by ECs may influence growth initiation and maintenance in earlier follicles even more so than more developed follicles. This finding further illustrates the importance of future studies geared towards learning EC influence on primordial follicle activation.

Observation of oocyte growth in addition to total follicle growth suggests that ECs may influence both oocyte growth and granulosa cell proliferation (Fig. 4a-b; Fig. 6). Higher estrogen production in co-cultured follicles provides an objective marker of granulosa cell function (Fig. 4c).

In identifying mechanisms by which ECs may contribute to improved survival and growth of early follicles, several angiocrines may be implicated, including VEGFA and Notch ligands Jagged 1, Dll 1 and Dll4. The importance of VEGF for survival and growth of early and advanced follicles has been reported [48]. Additionally, some suggest VEGF may inhibit apoptosis [49]. Presence of VEGF-A receptors on granulosa cells suggests the factor may promote proliferation events as well as play a potential role in primordial follicle activation in humans [50]. Interestingly, we noted that ECs derived from ovaries in the luteal phase expressed relatively more VEGF than the AOECs or fresh follicular ECs. This may be reflective of VEGF’s role in cyclic changes during formation and regression of the corpus luteum. Co-culturing early follicles with ovarian ECs provides this important angiocrine without need for supplementation.

Another potential mechanism to consider is interaction of follicular Notch with its ligands. There are four Notch receptors (Notch 1–4) and two families of ligands: Jagged 1, 2 and Delta-like (Dll) 1,3,4. Recent data shows that multiple Notch genes are expressed in early follicles and are regulated during the time of follicle formation [51]. In addition, attenuating Notch signaling by Notch signaling inhibitors decrease in-vitro follicular development [51, 52]. Lentivirus mediated overexpression of Notch intracellular domain 2 and c-myc could promote the proliferation of granulosa cells [52]. While roles for Notch in regulating folliculogenesis are beginning to emerge from mouse genetic models [53], the influence of extra-follicular exposure to Notch ligands on the follicle and its role in in-vitro growth and maintenance of isolated early follicles remains unknown. We show that AOECs express Jagged-1, Dll4 and Dll1 mRNA at a comparable level as fresh ovarian ECs. Therefore, we suggest that activation of Notch may contribute to the maintained growth and survival of early co-cultured follicles.

## Conclusions

We have established a novel platform that allows study of follicle-endothelial cell communication. Furthermore, we have demonstrated that co-culture with activated ovarian endothelial cells enables culture of primary and early secondary mouse ovarian follicles. This novel culture system not only adds to the growing number of described in vitro maturation systems, but also may facilitate the identification of factors that promote early follicular growth, potentially leading to new strategies for fertility preservation.
